# Use of home remedies: a cross-sectional survey of patients in Germany

**DOI:** 10.1186/1471-2296-15-116

**Published:** 2014-06-11

**Authors:** Lisa Maria Parisius, Beate Stock-Schröer, Sarah Berger, Katja Hermann, Stefanie Joos

**Affiliations:** 1 Department of General Practice and Health Services Research, University Hospital Heidelberg, Voßstrasse 2, Heidelberg 69115, Germany; 2 Karl and Veronica Carstens-Foundation, Am Deimelsberg 36, Essen 45276, Germany

**Keywords:** Home remedies, Traditional medicine, General practice, Symptom management, Minor health problems

## Abstract

**Background:**

Reliable information regarding patient knowledge of home remedies and the types of health problems patients use them for is scarce. Nevertheless, anecdotal evidence indicates that home remedies are used by patients for managing minor health problems and that this can be sufficient for symptom management while the body recovers from minor health problems. The aim of the presented study was to explore patient use of home remedies in Germany.

**Methods:**

A questionnaire was developed and pretested in a pilot study phase. The revised questionnaire was comprised of questions about general knowledge and experienced efficiency of home remedies, the use of home remedies for common health problems and socio-demographic data. Patients were recruited via randomly selected addresses of general practitioners (GPs) in three regions of Germany (Heidelberg, Erfurt and Hanover and surrounding areas). The questionnaire was handed out in the waiting area of GP practices. The data was analyzed descriptively.

**Results:**

480 of 592 patients from 37 GP practices were included, according to a response rate of 81%. Based on the survey results, home remedies were widely known and used by about 80% of our respondents (on average 22 different home remedies were used per person). The most frequently used home remedies were steam-inhalation, hot lemon drink, honey, chamomile tea and chicken soup. 80% of respondents tried home remedies *before* pharmaceutical options. Information about home remedies was most commonly gained from family members, rather than from written guides, media or GPs.

**Conclusions:**

These results provide an initial overview on the use of home remedies from the patient’s perspective in a German context. Bearing in mind the high use of home remedies that was reported by patients in the study, it is highly likely that GPs in Germany may need to advise patients on their use of home remedies during consultations. To this end, given the scarcity of reliable information on home remedies, further research is needed.

## Background

The use of home remedies for symptom management of minor health complaints is an area of health care that to date has not been extensively researched. Regarding existing literature a large amount of lay information can be found, but scientific literature is scarce. Public opinion polls available to date in Germany show that about 50% of the population use home remedies [1,2] and one study indicated that patients wish to be informed on the use of home remedies by their GP as well [2]. In the course of consultations with patients for treatment of common health problems, GPs may need to advise on the use of home remedies, although typically very little attention is given to this area in medical training. This is similarly the case in GP vocational training. For example, the “Oxford Handbook for General Practice” makes no mention of home remedies [3]. On the other hand, three prominent German textbooks for general practitioners encourage GPs to be at least familiar with the subject of home remedies and in some cases even to recommend them to patients [4-6]. However, in a study by Stevenson et al., they report that in general, “GPs discussed their lack of confidence in advising about home remedies saying they were ‘undertrained in non-pharmacological alternatives’” [7]. To the authors’ knowledge, there are no scientific studies evaluating use of home remedies for symptom management by patients in Germany. There is a need to increase the availability of reliable information and evidence on “home remedies” for general practitioners.

When researching the subject of “home remedies”, an important first step is to have a formal definition of this concept in mind, as this term is in common use in everyday language and can mean different things to different people. As major developments in linguistic theory from 20^th^ century have instructed us, meaning in language is a social construction [8]. This in itself is strong evidence for the importance of making clear what definition is understood by the term “home remedies” when conducting research in this field. Based on our literature review, we encountered the term “home remedies” regularly, but found no coherent scientific definition. For example, in the study by Stevenson et al. on patient’s use of self-treatment [7], the term “home remedies” is used eight times although the authors provide no definition of what they meant by this term (it is possible these authors considered the meaning to be clear enough that it did not warrant definition). Nevertheless, it highlights a problem when conducting research in this field. The lack of clear definitions of key terms and the fact that there is little reputable published literature in the field of “home remedies”, as an aspect of patients’ symptom management of minor health problems, highlights on the one hand a gap in this body of knowledge, and on the other hand, possibly reflects the low importance that has been placed on this subject to date. Therefore, we used the following self-developed working definition for the purposes of this study:

Home remedies are simple measures of symptom management for minor health complaints. Examples can be teas, wet packs, foodstuffs, skin applications and baths. Information is often passed along from one generation to another. Excluded are medicinal products such as dietary supplements, over-the-counter drugs as well as herbal therapy products, chinese teas, homeopathic globuli, Schuessler salts and Bach flower remedies and the like.

As illustrated in the examples from our working definition, home remedies can be many types of products used for a range of purposes in the household. Due to the nature of the subject “home remedies” neither sales nor prescribing statistics are available as is the case with pharmaceutical products. The direct source of data on the use of home remedies is surveying potential users. For this reason, this study was undertaken to contribute evidence within the body of research focused on patients’ symptom management of minor health complaints. The aim was twofold: a) to explore which home remedies are used to which extent and b) to explore which home remedies are used to treat minor health complaints.

## Methods

We conducted a cross-sectional questionnaire study of patients visiting a GP. Data collection took place between March and June 2013 in waiting rooms of GP practices. Data was collected using a convenience sample in three different regions in Germany: Lower Saxony (urban and rural GP practices), Thuringia (urban and rural GP practices), and Baden-Wurttemberg (urban and rural GP practices). Participation in the survey was entirely voluntary, and by filling in the questionnaire, patients agreed with participation in the study. The following ethics committees stated that ethics approval was not necessary for this anonymous questionnaire study: the Medical Ethics Committee of the University Hospital of Heidelberg, and the Ethics Committee of the Medical Association of Lower Saxony and the Ethics Committee of the Medical Association of Thuringia (personal communication).

### The questionnaire

No validated questionnaires in this context were available. Consequently, a standardised questionnaire was developed based on available literature and available questionnaires about knowledge and use of complementary and alternative medicine, for example, a study among elder patients in rehabilitation centres and a study about patients using both complementary and conventional medicine and their information about their practitioners’ qualification [9,10].

The developed questionnaire consisted of four parts. The first part contains a list of 49 home remedies adapted from the textbook “Home remedies in modern medicine” [11]. The predefined list of home remedies contains five groups: teas, foodstuffs, wet packs, baths and skin applications. Survey participants indicated whether each of these home remedies was known or not known, or already used successfully or not successfully.

The second part of the questionnaire includes items about the use of home remedies in general. For example, whether home remedies are used regularly, why they are used, and in which cases, and reporting of “subjective” effects. Also, the questionnaire asks how patients act when concerned about a common health complaint and from where their knowledge about home remedies comes. All items in this section were measured on 5-point-Likert scales from “never/not at all” to “very often/frequently”.

The third part of the questionnaire contains open questions about frequent health problems as defined by the German federal government’s health monitoring statistics [12]. Patients were requested to write down which home remedy they would use to treat symptoms of a cold, diarrhoea, constipation, back pain, headache, sleeping disorder/nervousness, wound healing and cystitis.

The last part of the questionnaire contains socio-demographic questions. The questionnaire was initially piloted with 10 volunteers. Subsequently, in a focus group with 12 doctoral students, the questionnaire was also piloted and then immediately afterwards discussed in a cognitive debriefing process; after which modifications to improve understanding and clarity were made. The revised version was piloted with a group of 10 patients recruited in the waiting room of a GP practice. No modifications were found to be necessary and the revised version was implemented for this study.

### Participants and recruitment

Patients had to be 18 years or older to be included in the survey. GP practices from three regions in Germany were targeted. Region selection was convenience based with a location in the north, the south and the east chosen: the cities of Heidelberg (in southern Germany), Erfurt (in eastern Germany) and Hannover (in northern Germany) and surrounding semi-rural areas. Within these regions, GP practices were randomly selected by their addresses. In all regions, patients from GP practices in both urban and rural areas were recruited with a final sample size of 400 patients in 40 practices (10 patients per practice). Addresses were obtained from the websites of the regional associations of statutory health insurance physicians (*kassenärztliche Vereinigung*). 100 GPs in each region were contacted by mail; this was followed by a reminder telephone call if there was no reply. In cases of a positive answer, dates for data collection were arranged. Information sheets and questionnaires were made available to patients in the waiting room of participating GP practices, and they were asked if they were willing to participate in the study by the first author during morning and afternoon consulting hours. On rare occasions, when a patient was not able to read or write, they were assisted in filling in the questionnaire. Completed questionnaires were collected in a clearly marked box left in the waiting room.

### Data analysis

Questionnaires were reviewed for completeness. When more than a third of the questions were not answered, including the socio-demographic data, questionnaires were excluded from analysis.

A descriptive analysis was performed with all other questionnaires, even if some data was incomplete. On top of variables related to the survey questions, an additional variable was created for analysing, per respondent, the entire number of named home remedies that were mentioned in the questionnaire. For this purpose, we tallied up home remedies from our list provided in the questionnaire as well as those named by participants in answers to open-ended questions. Home remedies were only counted once. The overall number of home remedies is reported as mean and standard deviation. A rating list was created for the 15 most frequently used home remedies from the predefined list in the questionnaire. Participant answers from open-ended questions were also classed into our pre-defined major categories i.e. teas, wet packs, baths, foodstuffs, skin applications. The use of home remedies by participants, based on our provided list of home remedies in the first part of the questionnaire, is presented as frequencies. Single items of the questionnaire regarding use of home remedies are presented as frequencies and percentage in case of pre-defined major categories. ANOVA was used to detect differences in the amount of home remedies used between sex, age, regions and rural or urban area All answers to items measured in 5-point-Likert scales are presented in tables.

For statistical calculations, SPSS 21.0 and Excel 7 were used.

## Results

A total of 37 GP practices agreed to have the survey conducted in their waiting rooms (Heidelberg 12, Erfurt 11 and Hannover 14). 20 GP practices were successfully reached by mail and 17 in a second step by follow-up telephone calls. The response rate was 81%: of 592 potential participants, there were 480 willing respondents who completed questionnaires. 460 questionnaires were analysed after exclusion of 20 questionnaires due to incompleteness. About 66% of the participating patients were women and 34% men with a mean age of 50.7 years (min 18, max 92, standard deviation 18). The percentage of privately insured versus state insured patients taking part in this study reflects national statistics for the German population. For more detailed information about socio-demographic data, see Table 1.

**Table 1:** Demographics of all respondents

	**n**	**%**
**Sex**	460	
Female	305	66%
Male	155	34%
**Health insurance**	454	
Statutory	406	89%
Private	46	10%
Other	2	0%
**Region of inquiry**	460	
Heidelberg	145	31%
Erfurt	146	32%
Hanover	169	37%
**Area of inquiry (=location of GP practice)**	460	
Urban area	240	52%
Rural area	220	48%

### Reasons for the use of home remedies

42% (176 of 441) of our respondents used home remedies very regularly (26% very often, 16% often). Another 38% of the respondents indicated that they used them quite regularly. 20% answered that they would not use home remedies very often (10% not much, 10% not at all). For reasons as to why respondents used home remedies, see Table 2. 73% of our respondents indicated that they used home remedies because of good experiences with home remedies (49% very often and 24% often). Four out of five respondents indicated that they used home remedies to treat specific health complaints (54% very often and 25% often). Two thirds of respondents used home remedies because of a recommendation from someone. 58% indicated that they felt better after the use of home remedies (29% very often and 29% often). 42% (27% very often and 15% often) used home remedies for prevention, about 47% (32% very often, 15% often) to avoid the use of medical drugs and about 40% to avoid side effects of medical drugs (27% very often, 13% often). Only 9% of respondents declared that a reason for home remedy use was that nothing else would help (4% very often, 5% often). Significantly, half of the respondents stated that they had used home remedies to take more self-responsibility for their health (30% very often, 19% often).

**Table 2:** Reasons for the use of home remedies

	**Very often**	**Often**	**Partly**	**Not often**	**Not at all**	**Total**
	**%**	**%**	**%**	**%**	**%**	**N**
**I use home remedies, because**						
Of good experiences	49.0%	24.0%	20.1%	4.2	2.7	408
Of recommendation	40.6%	25.7%	21.3%	5.9%	6.6%	409
I can self-care	30.1%	18.6%	28.4%	9.1%	13.7%	408
I feel better after	28.7%	29.4%	39.0%	1.7%	1.2%	415
I have them in stock	28.6%	22.2%	27.6%	12.8%	8.9%	406
I always did that	26.6%	19.9%	30.3%	12.7%	10.7%	403
Cheaper than drugs	18.6%	14.7%	23.8%	17.4%	25.5%	408
Symptoms fade away	13.6%	15.3%	59.6%	7.5%	4.1%	413
Nothing else helps	4.0%	4.7%	31.8%	27.8%	31.8%	403
**I use home remedies**						
For certain health issues	54.3%	24.8%	16.8%	1.7%	2.4%	416
Before the use of pharmaceuticals	35.5%	21.1%	26.1%	9.4%	7.9%	417
To do without pharmaceuticals	31.7%	14.5%	26.4%	12.3%	15.0%	413
To prevent side effects	27.3%	12.7%	20.2%	19.0%	20.9%	411
Preventive	26.7%	14.6%	19.9%	18.2%	20.6%	412
In addition to pharmaceuticals	25.4%	21.1%	29.3%	8.5%	15.7%	413
When pharmaceuticals do not help	19.3%	12.0%	26.6%	21.2%	21.0%	410

This table shows answers from closed questions about reasons for the use of home remedies.

### Which home remedies are used to treat common health-issues?

From the list of home remedies that we provided in the questionnaire, on average, respondents indicated that they used 18 home remedies out of 49 (min 0, max 48, standard deviation 9,5). In the section of the questionnaire with open-ended questions, an overall number of 1927 home remedies were named by 389 patients, on average; this was four different home remedies per patient (min 0, max 30, standard deviation 4.5). Considering both categories together, on average, 22 home remedies had been used by respondents (min 0, max 72, standard deviation 12.5).

**Figure 1:**
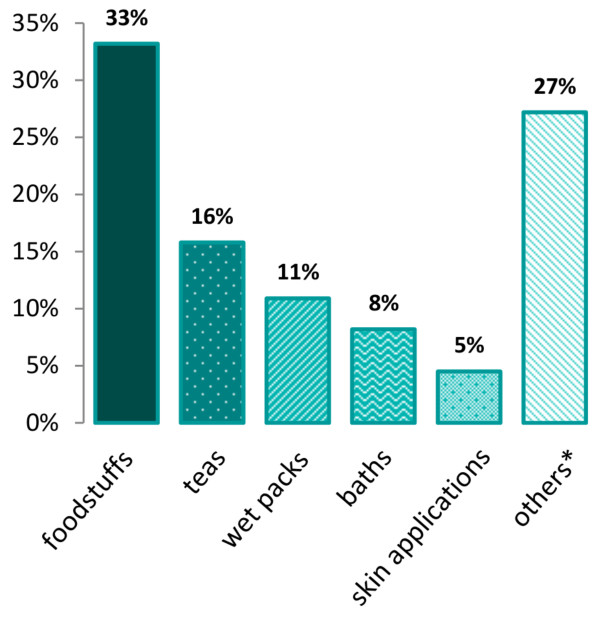
**Home remedy categories sorted according to their use of 100%.** *others: 15% of those 27% include ingredients as for example chamomile (3,6%), sage (2,9%), valerian (1,9%). As patients did not mention whether they would use it to eat, to drink as a wet pack or in a bath, it was not possible to categorise them. The other 6% were different home remedies, not often repeatedly named.

The most frequently used home remedies from the predefined list by our respondents were “inhalations”, “hot lemon drink”, “honey”, “chamomile tea” and “chicken soup”, see Table 3.Also, in the answers to the open-ended questions, inhalation and honey were found in the top five, followed by tea, chamomile, sage, salt and juice. Regarding potential categories, nutritional home remedies were most often used (33%), see Figure 1.

**Table 3:** Most frequently applied home remedies

	**Applying**	
	**N**	**%**
**Inhalation**	362	80%
**Hot lemon drink**	347	76%
**Honey**	346	76%
**Chamomile tea**	332	73%
**Chicken Soup**	330	72%
**Wheat sack**	296	65%
**Footbath**	291	65%
**Wet pack to calf**	278	62%
**Fennel tea**	270	59%
Chest rubs*	267	59%
**Sage tea**	268	58%
**Mint tea**	266	58%
**Steam bath**	236	52%
Back rubs*	230	51%
**Pretzel stick and coke**	229	50%

Answers from a predefined list of 49 home remedies sorted by most frequent applied home remedy by the total of all (N = 460) respondents.

*Meaning a home remedy is applied.

Not every common health complaint mentioned in the open-ended questions was treated by home remedies by every patient; however, our results indicate that respondents had definite preferences. Most frequently treated common health complaints were symptoms of a cold, more specifically: sore throat (74%), cough (72%) and runny/stuffed nose (65%), see in Table 4. In particular, for symptoms associated with respiratory infections, multiple home remedies were named by patients e.g. for runny/stuffed nose 15% (N = 67 of 460) named more than one home remedy, with the most frequent being steam inhalation, for cough 33% (N = 157 of 460) of respondents used a variety of different home remedies to treat the same symptom (e.g. drinking different teas, gargling and wet packs); for sore throat this was 27% (N = 122 of 460).

**Table 4:** Common health issues treated with home remedies

**Health issue/symptom**	**Patients symptom management with home remedies %**	
	**N**	**%**
**Sore throat**	338	**74%**
**Cough**	333	**72%**
**Runny/stuffed nose**	299	**65%**
**Diarrhoea**	245	**53%**
**Back pain**	209	**45%**
**Constipation**	174	**38%**
**Sleeping problems/Nervousness**	163	**35%**
**Cystitis**	155	**34%**
**Head ache**	136	**30%**
**Wounds/wound healing**	122	**27%**

This table records answers to open questions about the use of home remedies. It shows the most frequently treated minor health complaints with home remedies.

### Where does knowledge about home remedies come from?

**Figure 2:**
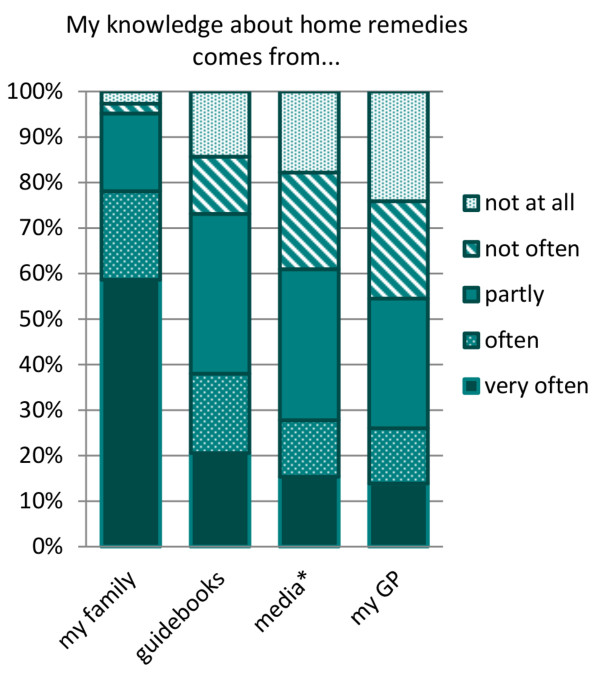
**Sources of knowledge about home remedies.** *media: internet, radio, television

Knowledge about home remedies in the respondent group came primarily from family members with 80% (N = 325 of 416). 40% (N = 157 of 413) consulted a guide book or magazine, 30% (N = 114 of 410) looked for information about home remedies in the media (including internet, radio and television) and 25% (N = 38 of 411) sought information from a GP, see Figure 2.

Statistically significant differences in the use of home remedies were identified. Women in this study used a larger variety of home remedies (p = 0,000) .There was a difference between the number of respondents that used home remedies who were located in Erfurt (in former East Germany) and those located in the region of Heidelberg (southern Germany). On average, respondents located in the region of Erfurt used almost five more home remedies per person than those in Heidelberg (p =0,003). There was no statistically significant difference in the use of home remedies according to age of the respondents nor between urban or rural setting.

## Discussion

### Home remedy use

In this survey, about 42% of the respondents indicated that they regularly use home remedies. The three most frequently used home remedies were: hot steam inhalation, hot lemon drink and honey in both categories i.e. the predefined list in the questionnaire and the open-ended questions. Nutritional-based home remedies were the most frequently cited type of home remedies used. Managing symptoms of a cold was the most common reason for using a home remedy. The majority of respondents in our survey preferred starting with home remedies to manage common health complaints and hoped to avoid the use of pharmaceutical drugs. Other reasons for home remedy use were good experiences with home remedies and alleviation of symptoms.

### Home remedies in other studies

During data collection, it was noted that certain interventions have not been taken into account as home remedies before because they are seen as everyday practices. Compared to public-opinion polls [1,2] and international studies including questions about the use of home remedies [7,13] the use of home remedies was far higher in our study. This might be explained by the fact that previous studies have included only a few questions about the use of home remedies. When the primary focus of research was, for example, patient-physician communication [7], self-care [14] or home remedy use as a part of CAM [13,15,16] not more than five different questions about home remedies were asked.

However, a 1990 study with 524 patients in Winnipeg, Canada [17] reports similar results to our study. Results from the Winnipeg study were close to our own in that the most frequently used home remedies were teas followed by honey, hot lemon drink, and hot steam inhalations. Again, similarly to results shown in our study, home remedies were used to treat common health complaints such as symptoms of a cold. In addition, in this study patients would recommend home remedies to others.

Another 1972 U.S. study included home remedies for symptom management and reported a 30% rate of symptom management by home remedies, with the most frequently used home remedies being bed rest, soaking and heating pads [18]. These home remedies, however appeared in only few of responses in our study, which might be explained by a different cultural context, a different time or a different research approach. A different cultural context might explain the significant difference of home remedy use in the Heidelberg and Erfurt regions as well, although we can only speculate on actual reasons.

### Reliable information on home remedies

Most symptoms of minor health complaints can be treated independently and, according to a UK population survey only a small percentage of these situations (12%) leads to a consultation with a GP [19]. Based on our results, respondents indicated that symptom management of minor health complaints with home remedies was experienced as predominantly positive. 97% of our respondents report that after use of a home remedy they felt better and symptoms decreased at least partly. We argue that those patients, who use home remedies as part of their symptom management for minor health complaints, are choosing to take a more active interest and role in their own health.

Patients taking an active role in their own health is an important aspect of modern medicine. As the 2007 *European Consumer Survey* highlights, patients wish to be advised on the use of home remedies [1]. Similarly, a 2003 German study, indicated that patients wish to receive advice on the use of home remedies [20]. Nevertheless, in our study, less patients gained information about home remedies from their GP than from family members. In the 2012 study by Joos et al., it was reported that surveyed patients considered that their GPs lacked knowledge about herbal remedies [21]. Although the article from Joos et al. is not specifically focussed on the topic of home remedies, it is possible to come to the conclusion that for similar reasons, patients are seeking information from family members (and elsewhere), rather than GPs, when it comes to home remedies. In addition to the information transmission from generation to generation or via media, GPs can (and we argue ought to) play a main role as a source of trusted health-related information, here including the use of home remedies [22].

From this discussion, there is clear evidence to support the fact that GPs need to be familiar with the subject of home remedies. Ideally, this would include which home remedies exist, which are beneficial and what risks need to be considered. Evidence shows that this is desired by patients and would support patients who wish to take a more active role in their own health.

Furthermore, there is a patient safety element that needs to be addressed, and GPs are in an ideal position, as trusted providers of health information, to provide patients with guidance. A few studies show that adverse effects or interactions may occur with other treatments, for example, possible interactions between garlic and cranberries with warfarin [23,24]. There are certainly a few studies to be found that describe interactions between herbal remedies and pharmaceutical products. Nevertheless, although “natural” these are “medicinal products” and fall outside the focus of this research according to our working definition of “home remedies” (see *Background* section). For this reason, such papers have not been included in this discussion, although we are aware of such studies.

It also means clinical research has to be increased to evaluate the efficacy of frequently used home remedies and to identify possible side effects of these interventions. Only a few clinical trials on specific home remedies have been conducted to date, such as about honey for use in wound healing [25]. However, because benefits and risks have not been evaluated for the majority of home remedies, it creates an on-going difficulty for GPs to access reliable information and evidence on home remedies.

### Strengths and limitations

To minimize the selection bias, every patient in the waiting area of the participating GP practices was invited to participate in the study; invitations were not based on whether they knew about, or used home remedies. As there is no known “scientific” definition for home remedies, based on information from a textbook, we designed a predefined list of home remedies and included this in the questionnaire. It cannot be excluded that patients could have been thereby influenced. The examples given might have led to an increase in the amount of indicated home remedies used, where everyday practices may not have been understood as home remedies before reading the questionnaire. Furthermore, answers to the open-ended questions did have a wider range of expressions than found in our predefined home remedies list. Therefore, these answers were also classed into the overarching categories of: teas, foodstuffs, wet packs, baths, and skin applications. A problem encountered with the use of a five-point Likert-scale for some of our questions, was that the response “partly” in the context of home remedy questions could have been understood as partly=”sometimes” or partly=”concerning certain home remedies”. Both types of answers must be considered regarding the results.

## Conclusion

This study is a first survey giving an initial overview on the use of home remedies from the patient’s perspective in Germany. Bearing in mind the indicated high use of home remedies for symptom management of minor health complaints, it is highly likely that GPs may need to advise on the use of home remedies in consultations. There is anecdotal evidence on the use of home remedies, based on personal experiences and recommendations, however, GPs need access to reliable information concerning health risks and benefits. There are also benefits to be gained in terms of supporting patients to take an active role in their health, when GPs are able to provide advice on the use of home remedies and incorporate possible home remedies into symptom management plans for minor health complaints, as many patients state they desire. Nevertheless, it cannot be ignored that there is a significant gap in the body of medical knowledge on the subject of home remedies, and although it reflects the low importance that has been placed on this subject to date, given the amount of indicated use by patients and regional differences, further scientific research in this area needs to be conducted.

## Competing interests

The authors declare that they have no competing interests.

## Authors’ contributions

LMP has made contributions to conception and design of the study, did the data collection, performed the statistical analysis and interpretation of the data, did the drafting and revising of the manuscript. SJ and BS-S have made contributions to conception and design of the study. SB participated in drafting and revising the article. SJ has given final approval of the questionnaire and been involved in revising the manuscript critically for important intellectual content. KH has made contributions to the statistical analysis and interpretation of the data. All authors read and approved the final manuscript.

## Pre-publication history

The pre-publication history for this paper can be accessed here:

http://www.biomedcentral.com/1471-2296/15/116/prepub
